# Stroke survivors', caregivers' and GPs' attitudes towards a polypill for the secondary prevention of stroke: a qualitative interview study

**DOI:** 10.1136/bmjopen-2015-010458

**Published:** 2016-05-13

**Authors:** James Jamison, Jonathan Graffy, Ricky Mullis, Jonathan Mant, Stephen Sutton

**Affiliations:** 1 Behavioural Science Group, Primary Care Unit, Institute of Public Health, University of Cambridge School of Clinical Medicine, Cambridge, UK; 2 Primary Care Unit, Institute of Public Health, University of Cambridge School of Clinical Medicine, Cambridge, UK; 3 Primary Care Unit, University of Cambridge, Strangeways Research Laboratory, Cambridge, UK

**Keywords:** STROKE MEDICINE, PRIMARY CARE, QUALITATIVE RESEARCH

## Abstract

**Objectives:**

To understand the perspectives of stroke survivors, caregivers and general practitioners (GPs) on a polypill approach, consisting of blood pressure and cholesterol-lowering therapies, with or without aspirin, for the secondary prevention of stroke.

**Methods:**

A qualitative interview study was undertaken in 5 GP surgeries in the East of England. 28 survivors of stroke/transient ischaemic attack (TIA) were interviewed, 14 of them with a caregiver present, along with a convenience sample of 5 GPs, to assess attitudes towards a polypill and future use. Topic guides explored participants attitudes, potential uptake and long-term use, management of polypill medication and factors influencing the decision to prescribe. Data were analysed using a grounded theory approach. Key themes are presented and illustrated with verbatim quotes.

**Results:**

The analysis identified 3 key themes: polypill benefits, polypill concerns and polypill lessons for implementation. Stroke/TIA survivors were positive about the polypill concept and considered it acceptable in the secondary prevention of stroke. Perceived benefits of a polypill included convenience resulting in improved adherence and reduced burden of treatment. Caregivers felt that a polypill would improve medication-taking practices, and GPs were open to prescribing it to those at increased cardiovascular risk. However, concerns raised included whether a polypill provided equivalent therapeutic benefit, side effects through combining medications, consequences of non-adherence, lack of flexibility in regulating dosage, disruption to current treatment and suitability to the wider stroke population.

**Conclusions:**

Participants acknowledged potential advantages in a polypill approach for secondary prevention of stroke; however, significant concerns remain. Further research on the efficacy of a polypill is needed to reassure practitioners whose concerns around inflexibility and treatment suitability are likely to influence the decision to prescribe a polypill for secondary prevention of stroke. Acceptability among survivors, caregivers and GPs is likely to determine the uptake and subsequent use of a polypill in the future.

**Strengths and limitations of this study**

- This research adds to an important body of work exploring cardiovascular polypills and is the first study to focus on attitudes to a polypill for secondary prevention of stroke.
- The findings are strengthened by the inclusion of caregivers who have an important role to play in managing the medication of stroke/transient ischaemic attack survivors.
- Conducting a qualitative assessment of individual perspectives allowed an in-depth examination of the subject area.
- Owing to the limited sample size, findings may not generalise to the wider stroke population or necessarily represent the views of all general practitioners.
- Future research should consider harder to reach groups such as those who need support to manage medication and may benefit most from a polypill approach.

## Introduction

Stroke is the fourth most common cause of death in the UK, responsible for ∼40 000 deaths every year^1^ and is also a significant cause of acquired adult disability,^2^ with about half of all survivors experiencing some degree of physical or cognitive impairment^3^ and left dependent on others.^4^

People who have had a stroke or a transient ischaemic attack (TIA; also known as a ministroke) are at higher long-term risk and therefore exposed to the increased possibility of having a further event.^5^ ^6^ However, this risk can be substantially reduced through the use of preventative medications such as antiplatelet agents^7^ or anticoagulants,^8^ as well as cholesterol-lowering^9^ ^10^ and blood pressure (BP)-lowering therapies.^11^

Despite evidence-based guidelines, treatment for stroke often falls below recommended standards,^12^ ^13^ and significant deficiencies in secondary prevention care have been reported.^14^ The use of multiple medications to treat cardiovascular disease (CVD) is often associated with inappropriate medication use (eg, underuse, or use of non-appropriate medicines), underprescription and reduced adherence.^15^ A polypill consisting of cholesterol-lowering and BP-lowering therapies, with or without aspirin in a single pill for the treatment of CVD^16^ has been proposed.

Wald and Law^17^ introduced the polypill concept and estimated a theoretical 88% reduction in ischaemic heart disease and 80% reduction in stroke, if taken by everyone over 55 years. Since then a growing body of literature has developed around a polypill, otherwise known as a fixed-dose combination (FDC) pill, for the prevention of CVD.^18^ ^19^ A series of recently completed trials investigating the role of a FDC pill on adherence to medication for secondary prevention demonstrated improved adherence for the polypill strategy compared with standard care.^20–22^ Elsewhere, FOCUS found improved adherence for patients with myocardial infarction in the polypill group compared with the group given the three drugs separately.^23^

To date, a small number of studies have investigated the perspectives of patients and healthcare professionals towards a theoretical polypill. Although cardiovascular patients considered it convenient, they had concerns around the inflexibility of a polypill^24^; however, general practitioners (GPs) would consider prescribing it to those who needed secondary prevention medication if it was shown to be effective.^25–28^ With adherence to medication in stroke survivors known to be suboptimal,^29^ this group may be particularly suited to treatment with an FDC polypill.

The aim of this study was to explore the attitudes and perspectives of stroke/TIA survivors, carers and GPs towards a polypill approach for the secondary prevention of stroke, including the benefits and consequences of using a polypill, factors likely to influence uptake, the caregiver role in managing medication, and GPs' views and attitudes towards prescribing a polypill in the future.

## Methods

### Study design and participants

A qualitative study using semistructured interviews was undertaken. The stroke registers of five GP practices in the East of England were searched. The criteria for inclusion of stroke survivors was being over the age of 55 years, with a diagnosis of stroke or TIA and able to speak English. Based on these criteria, a list of prospective participants was generated by the practice administrator. The list was then screened by the practice GP and anyone deemed unsuitable, such as those unable to provide informed consent or who were terminally or seriously ill, was removed. Purposive sampling was used to recruit stroke/TIA survivors with maximum variation characteristics representing a spread of socioeconomic status,^30^ age, gender and disability.^31^ Survivors were sent a study information pack and invited to interview. Caregivers were approached by the stroke survivor with a study information pack and invited to participate. All caregivers were subsequently interviewed in the presence of a stroke survivor. Owing to time constraints, we chose not to interview caregivers separately. All interviews were conducted in the stroke survivors' homes. We also sought the views of a convenience sample of GPs, each of whom was the study lead for their practice. The GP was contacted by phone and an interview arranged at their place of work. The number of interviews conducted was determined by data saturation, the point where no new information emerged from discussions. Interviews were face to face and consent was taken in person before any discussion started.

### Data collection

Data were collected through semistructured interviews with open-ended questions that defined the area to be explored.^32^ Topic guides were developed by the authors and informed by current literature in the field and expertise within the study team which included a GP, a qualitative researcher and a stroke expert. To ensure ease of understanding and suitability, topic guides were piloted with two stroke survivors and checked by a GP. Any appropriate recommendations were considered and implemented. Data from the two pilot interviews was included in the final analysis. All interviews were conducted by the lead author (JJ) who has considerable experience in qualitative research analysis. Field notes were also taken by the interviewer. Topics discussed were perceived benefits and consequences of a polypill, factors influencing polypill uptake, caregiver views, and GPs' beliefs and attitudes towards prescribing a polypill (see online supplementary file 1 for the interview schedule). The schedule of questions was refined and finalised after the fifth interview to include questions on the wider experience of stroke as well as understanding of the polypill approach and the GP relationship. Interviews were audiotaped, lasted 1–1.5 h and were transcribed verbatim.

10.1136/bmjopen-2015-010458.supp1 Supplementary data

### Data analysis

We followed the Strauss and Corbin Grounded Theory approach using constant comparative analysis.^33^ This method permits key points to emerge from the data and to then be coded individually. A set of codes, representing initial themes, were developed from chunks of data. Codes were then further refined, and those representing similar concepts were grouped together to form categories. The identification and refinement of categories continued until the final themes emerged. Nvivo V.9 (QSR Intl, Melbourne, Victoria, Australia) was used to organise, code and manage the data. Transcripts were entered into the program and coded by JJ, with 20% double coded independently by SS. Queries arising from coded transcripts were settled through discussion. Communication with a third author (JG) enabled clarification and refinement of categories until a consensus was reached.

## Results

A total of 28 stroke/TIA survivors participated. Fourteen were interviewed alone and 14 with the caregiver present, who was either a spouse (n=12) or family member (n=2). Characteristics of survivors are displayed in table 1. Three male GPs and two female GPs were also interviewed. One GP was white British, one was Chinese and three were of south Asian origin. Key themes identified reflected the positive and negative aspects of the polypill approach as well as future use. Subthemes highlighted benefits and concerns associated with a polypill approach and factors likely to influence stroke survivors using a polypill.

**Table 1:** Stroke survivor characteristics

Gender	Male: n=21 (75%) Female: n=7 (25%)
Age (mean: 74 years)	60–69 years: n=10 (36%) 70–79 years: n=11 (39%) 80–89 years: n=7 (25%)
Ethnicity	White: n=27 (97%), South Asian: n=1 (3%)
Stroke classification	Stroke: n=14 (50%) TIA: n=14 (50%)
Time since stroke	6 months to 2 years; n=10 (35%) 3–5 years: n=8 (29%) 6–10 years: n=5 (18%) >10 years: n=5 (18%)
Diabetes status	Yes: n=9 (32%) No: n=19 (68%)
Smoking status	Non-smoker: n=15 (54%) Ex-smoker: n=11 (39%) Smoker: n=2 (7%)
Interview status	Survivor and caregiver: n=14 (50%) Survivor only: n=14 (50%)
Rankin score* MrS-9Q	No symptoms: (0) n=6 (21%) No significant disability: (1) n=4 (14%) Slight disability: (2) n=6 (21%) Moderate disability: (3) n=4 (14%) Mod severe/severe disability: (4–5) n=8 (29%)

*Rankin score is derived from a scale that measures the degree of disability in the daily activities of people who may have suffered a stroke.

MrS-9Q, Modified Rankin Scale; TIA, transient ischaemic attack.

### Polypill benefits

The concept of a polypill was broadly acceptable to survivors and caregivers. Greater convenience leading to better adherence, confidence that a polypill was providing the appropriate treatment, reduced treatment burden, ease of use and improved medication management were all considered benefits. For GPs, a polypill facilitated medication taking and provided flexibility in treatment and convenience around prescribing practices.

#### Convenience

Survivors were enthusiastic about one tablet combining all stroke medication and reducing treatment burden through minimising the inconvenience of managing multiple medications.

That is the best thing I've read when it said you might have to take one pill to cover the lot. Super, because that is just a bugbear, it's a bugbear in life. (p. 11, Male, 73 years)

A single tablet was considered easier to remember and likely to improve overall medication-taking behaviour.

I think it's brilliant because erm I, I've got more chance of remembering to take one tablet than I have of remembering two different times of the day if you like. (p. 10, Male, 66 years)

Caregivers also endorsed the view that a polypill improved compliance and that it ensured the appropriate medications were being taken.

It means that if you've taken that one you've taken them all. Whereas sometimes if you run short, you think oh I'll just take that one and forget about the other one until you go to the doctors and get the refill. (p. 02, Female, carer)

GPs also felt that a polypill had the potential to improve medication adherence.

I think that would reduce the pill burden to our patients and I think that's very good idea…I think he would be very compliant with it, because he is thinking that he is going to be taking 1 tablet and not 5 tablets…(GP 02, Female)

The potential for ‘cross-over’ treatment in individuals with multiple existing cardiovascular comorbidities was mentioned.

If you're giving polypill in the form of one pill, even with people with comorbidities (you're) maybe reducing their number…and might improve overall compliance and it may have the side effect of improving their comorbidity as well. (GP 05, Male)

For carers, the polypill approach made the medication-taking process less demanding.

It's logic to me and I think it's an excellent idea if it could be done, certainly instead of *[patient]* fiddling about in a saucer trying to pick up tablets. (p. 28, Male, carer)

They also felt that the process of managing medication was better, compared with using multiple medications.

Well if it's only one tablet a day it would be quicker, wouldn't it? for a start. I mean I usually sit on a night-time and do that (pillbox) when I'm watching telly. There's a few times I've missed out the odd tablet or put a double in or put too many in so I mean that would be easier. (p. 02, Female, carer)

#### Benefits of correct treatment

A polypill offered the benefit of correct medication and it ensured that the patient received their recommended medications.

It could protect, once you had polypills that contained a mixture of medications which are known not to have…contradictory side-effects…then you would feel very safe. (p. 03, Male, 86 years)

And as long as it's whether it's one pill or four pills so you know this is my point of view I don't think it's going to affect I mean other people might oh yeah I could have four pills instead of one and they'll start worrying about it but no I erm I just accept that, that the people are doing their job properly and getting their facts right…as I say as long as the scientists have got it alright you know you've got to have faith in them. (p. 08, Male, 87 years)

There was also confidence that components were safe, tested and therefore provided the most appropriate treatment.

I'm all for these things…it might not be good for you, It might not, I don't know I can't see how because if they're now gonna put four different pills into one they musta investigated a, b, c and d to put them in one so therefore it's going to be beneficial to me and anybody else that wants those four in one. (p. 11, Male, 73 years)

### Polypill concerns

Survivors' and caregivers' concerns included polypill non-compliance resulting in missing all medications, inability to adjust dosage, whether a polypill could maintain the benefits of the survivors' current secondary prevention medication, timing of a polypill, identifying the source of polypill side effects and modifying treatment if a component was no longer required. GPs questioned whether a single pill could treat the entire stroke population, the cost implications of treatment and the wisdom in modifying a patient's stable treatment regimen.

#### Appropriateness of treatment

Several survivors expressed concern that a polypill may not sustain equivalent therapeutic benefit of secondary prevention treatment.

As far as I'm concerned you've got one tablet with all the ingredients of the others…if I've got the same erm dosage of statin and if it didn't disturb my readings then yeah I mean erm what are the objections to it? (p. 05, Male, 64 years)

Others also had concerns about the prospect of a ‘pill for all’, inability to alter dosage and being less amenable to dose titration, if that was required.

Would the polypill be in different strengths because like for blood pressure at the moment I'm taking…12 and a half, and then me cl- clopridogrel is 75…maybe six months down the line my blood pressure can reduce, what would that do with the polypill? (p. 21, Female, 68 years)

Survivors accustomed to scheduled medication regimens also questioned how drugs could now be combined and taken at a single time point.

if you've got them altogether and you're supposed to take those tablets at different times of the day, how's it going to work? Is it going to upset your system? (p. 22, Female, 71 years)

#### Suitability of the polypill strategy

Survivors questioned the ease of managing treatment if one or more components were no longer required.

Would it only be suitable for somebody who's taking four of that particular medication? But what would happen if say the Dr said, you're not so bad so you don't need to take that particular tablet? (p. 16, Female, 82 years)

A few expressed concerns around the inclusion of statins in any combination pill.

Yes has that got anything to do with statins? I've read a lot about statins and I'm afraid I feel I wouldn't want to take them. Because the side effects and everything. (p. 19, Female, carer)

GPs were cautious, suggesting a polypill could be better suited to those on similar medications whose treatment was well established.

I think the right drugs in the right combinations there, it, would potentially be helpful for a cohort of people. I don't think it will be for everyone but there will be a cohort of people who will probably be on very similar drugs…(GP03, Male)

Survivors and carers were also concerned that poor adherence would lead to missing all their secondary prevention drugs.

If you’re gonna give them a polypill that is three or four tablets and they don't bother taking that…They're gonna be worse off. (p. 14, Male, carer)

Given the unique needs of stroke survivors, some suggested that multiple polypills may be needed.

They don't give me three separate ones for no reason, there must be a reason for it. You can’t do that with a polypill unless you have a hundred polypills all different medications and different combinations. (p. 18, Male, 88 years)

#### Polypill side effects

The likelihood of polypill side effects led many to question the suitability of single pill treatment.

The fine tuning takes a bit of doing so w- with the one pill I got my bit of a doubt that it might work for some people but it might not work for everybody you see. (p. 04, Male, 80 years)

For GPs, a further problem resulting from this was the potential difficulty in identifying the component of a polypill responsible for side effects.

My personal anxiety is about side effects when you club two, three medicines together, if one of them, one of the components is, is causing the side effect then you'll not know, you may have to again change. (GP 05, Male)

#### Medication adjustment

GPs questioned the benefit in altering established medication routines to accommodate a polypill in those who were already taking their medication as directed.

If you've got, as I said, a very motivated patient they are happy with what they are taking, then we don't probably have to intervene, but we may have to give to people who are not that motivated or compliant. (GP 05, Male)

They also expressed concern about the inconvenience of having to readjust future treatment if polypill components were no longer required.

If somebody has a problem ok well we'll just stop using the polypill and give them the individual ones but with that stopping and chopping and changing people will say they've changed my tablets again, that becomes an issue. (GP 04, Male)

However, inflexibility of a polypill and the inability to manipulate dosage was perhaps the greatest concern among GPs.

We do switch around quite a bit different brands, different sizes, statins and sometimes it may not be the right dose but you kind of slowly edge it in…It would be advantageous if it was a single pill but that would be maybe a bit difficult with polypill…It's the fine tuning that's difficult. (GP 01, Female)

Caregivers also expressed concern around the inflexibility of a polypill and the potential difficulties in adjusting dosage.

You would have to get the right strengths of each tablet. “Where you were on atenolol 50 you are now on 25”. Sometimes they change the strength of the tablet. That's where it would be harder to change with the polypill. (p. 25, Female, carer)

#### Size of polypill

GPs raised concerns that a large pill could actually discourage medication taking.

Yeah is it a horse tablet?…that's going to have the other, the opposite effect on compliance that we want…People are going to start breaking it having half now and half twelve hours later. (GP 03, Male)

The size was also highlighted by caregivers who expressed concerns around a prospective polypill being very large.

Not going to be horse pills are they…as we call them, 500 mg. (p. 07, Female, carer)

For some stroke survivors, a single pill was considered much easier given the potential problems associated with multiple medications which could be larger and more difficult to swallow.

If you can get it into one, it's so much better, you haven't got to put all these tablets down your throat. I mean like this might get stuck, and one of my tablets, if it gets stuck it burns my throat so much so the other week I lost my voice. (p. 06, Male, 61 years)

#### Cost of polypill

The burden of the polypill on National Health Service (NHS) resources was also raised with a number of GPs suggesting that a more expensive pill could be difficult to prescribe.

If it is cheaper then there won't be an issue at all. if it comes out to be more expensive than the four tablets which you are giving individually to the patient then it comes to be an issue. (GP 02, Female)

Cost implications for practices and pharmacies dispensing a polypill were also considered with GPs acknowledging the likelihood of reduced revenues associated with a single pill.

They get an item fee for each thing they prescribe so if you have 4 drugs you get a fee for each, if you put it in 1 pill that will account for one. (GP 04, Male)

### Polypill lessons for implementation

Survivors thought that whether they used a polypill in the future would depend on their doctor's recommendation, but they also questioned the need for a polypill given their satisfaction with current treatment. GPs acknowledged that their support was likely to be influential in the decision to use a polypill and believed the approach should be adopted if it was found to be beneficial to the patient. While stroke/TIA survivors were generally positive about the polypill approach, many were non-committal on its future use, largely due to the lack of existing evidence.

#### Polypill recommendation

Caregivers felt that whether they used a polypill in the future was likely to depend on their doctor recommending the treatment.

It sounds good but w- we've got to, we would have to weigh up, listen to what the doctors say and the consultants and see what history, because this polypill, from what we've hear. Very, very little, it's quite new, that's all we know. (p. 22, Male, carer)

While GPs felt comfortable with the polypill approach, there was a preference for recommending a polypill to those who were already using the medication components.

I don't think I'd be comfortable saying here's a new stroke patient, just start them with a polypill as a starting point, I think I'd feel uncomfortable with that.

If I had patients that are on the four drugs that are in there erm I think I'd probably feel fairly comfortable saying well here's one tablet that's got all of those things you’re on already. (GP 04, Male)

#### Satisfied with current medication

Being content with their current medication also made survivors less enthusiastic about taking a polypill which may have unwanted side effects.

Why take a tablet that perhaps will affect you. Plus the fact I'm perfectly happy with what I'm on, you know, at the moment anyway. Perhaps if I go a bit doo-lally or you know erm…I would consider it. (p. 01, Female, 71 years)

While a concern raised among some study participants was that there was as yet little scientific evidence in support of a polypill approach.

No, I don't think I'd like to be a guinea pig with it though…I don't know, I think I would rather continue with what I've got until it's absolutely perfected the polypill. Get somebody else. (p. 23, Female, 74 years)

#### Endorsement of the polypill

GPs agreed that if they endorsed polypill, stroke/TIA survivors were likely to accept it as a treatment for secondary stroke and commit to using it in the future.

I think the majority of our current patients if we told them we think this is the right thing to do would probably be happy with that. It's a fairly easy argument. (GP 03, Male)

Furthermore, there was an obligation to try new and innovative treatments like the polypill, if its potential benefits were proven.

I welcome change and innovation I'm excited by it…you don't know until you've tried it…We have to try it if there was a potential benefit there for people. (GP04, Male)

## Discussion

### Summary of main findings

Stroke/TIA survivors and caregivers felt a polypill offered greater convenience, reduced the burden of treatment and improved adherence to medication. A polypill also ensured that patients received the correct treatment and that medications were safe. However, survivors expressed significant concerns around the suitability of a polypill if not already using its individual components, the size of a polypill and the implication for using a polypill if any component was no longer needed. Other important limitations identified by participants included the potential for side effects and the inflexibility of the single pill approach. GPs felt that a more expensive pill would be problematic and acknowledged that their endorsement was key to it being accepted. For survivors, the decision to use a polypill would depend on the GP's recommendation, but those who were satisfied with their current treatment regimen felt less inclined to change to a polypill.

### Strengths and limitations

A strength of this study is that it adds to a growing and important body of research on attitudes towards a cardiovascular polypill with a focus on secondary prevention of stroke. Second, the use of semistructured interviews enabled an in-depth assessment of individual perspectives. A further strength is the inclusion of caregivers, who can make a significant contribution in the future management of polypill treatment. We believe that being interviewed by a qualitative researcher rather than a healthcare professional encouraged survivors to be more open and to engage in discussion.

However, limitations include a relatively small sample of GPs recruited from five general practice surgeries. Although every effort was made to recruit a representative sample with varied disability, most survivors who responded to our request to participate were primarily able bodied with no significant stroke symptoms and independently managed their own medication. In addition, survivors were almost exclusively white British. With some ethnic groups, particularly south Asians, known to be at considerably higher risk of CVD,^34^ the study may have benefited from the including individuals who are considered to be at a greater risk from stroke and likely to be prospective users of polypill therapy. As a result, survivors in our study may not represent the wider stroke population. Furthermore, only five GPs were interviewed, and their opinions may not reflect those of the GP population at large. With all caregivers interviewed in the presence of a survivor, this may have contributed to individuals responding in a socially desirable manner and understating their true views on secondary prevention and the polypill. Investigating a polypill among survivors with significant symptoms and dependent on others to organise their tablets may be an area for future research in the field. Finally, future research should aim to include those harder to reach groups of survivors who may benefit most from a polypill approach.

### Comparisons with existing literature

The inflexibility of treatment and the potential for side effects were considered key challenges of a polypill approach. Concerns about side effects have previously been identified as influencing medication-taking behaviour^35^ and recognised as a significant barrier to adherence in CVD medication.^36^ Our findings are also in line with a recent UK primary care investigation in which patients considered a secondary prevention polypill acceptable, but were concerned about components interacting and inflexibility of treatment.^37^ The inability to adapt polypill dosage and the suitability of fixed dose treatment was a key concern for GPs in our study and has been previously reported in studies exploring polypill attitudes among GPs elsewhere. A small survey of 17 practitioners in New Zealand reported that having no choice of polypill components or doses was the thing GPs disliked most about the concept of a polypill.^25^ In another UK study of primary healthcare professionals, inability to titrate dosage was considered a major disadvantage of the polypill.^28^

The GPs in our study agreed that cost was a potential impediment to prescribing a polypill in the future. Compared with free combination medications, FDC therapy has the potential to be relatively inexpensive due to cheaper drug costs and reduced monitoring,^38^ and there is increasing evidence in the literature supporting the cost-effectiveness of a polypill strategy.^39^ ^40^ With modest costs considered a cornerstone of combination therapy,^41^ evaluations of the cost-effectiveness of using polypills is urgently needed.

Improved adherence was recognised as a key advantage of a polypill, and survivors acknowledged that a single medication episode was easier to remember. With frequent dosing regimens^42^ and polypharmacy associated with poor patient compliance to cardiovascular medications,^43^ ^44^ a polypill approach offering a simplified medication regimen has the potential to improve adherence in the treatment of CVD.^24^ ^45^ Our study corroborates observations from a patient perspective on whether a polypill could improve adherence, which highlighted concerns around the efficacy of a polypill compared with current medications and the potential for side effects.^24^

For caregivers, benefits of a polypill included simplifying the medication-taking process and ease in organising pill boxes. In a recent study on factors that influenced caregiving and medication management, participants recognised complex medication needs as an impediment to care by increasing the demands placed on the caregiver.^46^ Caregivers in our study recognised that a polypill approach was potentially more convenient for the pharmacy, an observation which has been confirmed in a recent qualitative investigation exploring pharmacists' views towards a cardiovascular polypill.^47^

Stroke/TIA survivors expressed a reluctance to adopt a future polypill strategy, citing GP approval as a key factor. This not only supports the view that cardiovascular patients were inclined to do what their GPs told them^48^ but also highlights the key role GPs can play in promoting a polypill approach. Exploring the perspectives of those with direct experience of the polypill can contribute to the wider acceptability of a polypill strategy and should continue to be a priority of future research. While a polypill was acceptable to most patients of the UMPIRE trial, some felt that FDC therapy was less tailored to individual patient needs.^49^ A recent investigation of the views of cardiovascular patients and providers who participated in polypill trials reported similar advantages and concerns to those identified in our study,^50^ suggesting that polypill perspectives translate to other regions and healthcare settings.

With research suggesting that health practitioners often fail to fully explain the important elements of medication when first prescribing treatment,^51^ uptake of a polypill may depend not only on the GP prescribing therapy but also on informing and encouraging acceptance of the approach among stroke/TIA survivors and their caregivers.

### Implications for clinical practice

This study identified some positive aspects of a cardiovascular polypill for the secondary prevention of stroke. However, greater efforts are needed within the clinical practice setting to reassure patients of the benefits of a polypill. Health professionals’ endorsement when prescribing a polypill could also lead to greater acceptance of this treatment approach and its use among stroke survivors, particularly as inadequate information and difficulties with new medications are associated with poor adherence.^52^ Further studies are needed with a broader sample of GPs to corroborate the findings reported here. With adherence among stroke survivors known to be suboptimal,^29^ this patient group may be particularly suited to receiving treatment using FDC polypill therapy. Further research on the efficacy of a polypill will also reassure practitioners whose concerns around inflexibility and the suitability of treatment are likely to influence the decision to prescribe a polypill to stroke/TIA survivors.

## Conclusion

A growing body of evidence suggests that a FDC pill may have a role to play in the prevention of CVD. This study contributes to the growing literature on cardiovascular polypills, offers a unique insight into the field of stroke, and may inform future research and clinical practice on secondary prevention in the UK. A polypill may also have a role to play in improving adherence among stroke survivors. The findings have informed the development of PROPS—Preventative Role of a FDC Pill in Stroke—a multicentre, open-label, randomised controlled trial of a FDC pill versus standard care for secondary prevention of stroke in a primary care setting (EudraCT number: 201300472229). However, addressing patients' and practitioners' concerns and intensifying efforts to increase the acceptability of this treatment approach is likely to determine future use of a cardiovascular polypill for the secondary prevention of stroke.
