# Supplementary material for: Stroke survivors', caregivers' and GPs' attitudes towards a polypill for the secondary prevention of stroke: a qualitative interview study
Source: BMJ Open. 2016 May 13;6(5):e010458. doi: 10.1136/bmjopen-2015-010458 (PMC4874103; doi:10.1136/bmjopen-2015-010458)
Supplement: Supplementary data [file bmjopen-2015-010458supp.pdf]

Supplementary File 1

Topic guide for semi-structured interviews

Stroke survivors

Can you tell me a bit about your experience of having a stroke?
What were you advised to do? What do you know about the risk factors for having another stroke?

Taking your medication?
Can you tell me about your current medication taking experience? Anydifficulties?

Do you know what a polypill is?
What do you think of being able to take a single pill (containing a combination of different stroke medications in one pill) instead of your usual medication?
How do you think this would change the experience of taking medication?
What would you consider to be the advantages of taking a polypill?
Can you think of any reasons why taking a polypill might not be a good thing?
Would you consider taking a polypill in the future?

Can you tell me about your relationship with your GP?

Carers

Can you tell me about your experience of being a carer?
How informed do you feel you are? about your stroke survivors condition?

Do you manage the medication? Can you tell me about this?
Are there any specific difficulties related to patient taking the stroke medication?
Can you think of any ways in which the medication taking process could be made easier/improved?

Have you heard of a polypill?
What do you think of the idea of a 'polypill'
What do you think of the patient taking a single polypill instead of their usual stroke medications?
How do you think this would benefit patient's medication taking behaviour?
Can you think of any reasons why taking a polypill may not be a good idea?
How do you think a polypill would enable better management of medication?
Can you think of any ways taking polypill would be a disadvantage?
What do you think about using a polypill in the future?

GPs

Current Practice
Can you tell me about current practice for secondary prevention of stroke?
Can you think of any limitations associated with current practice?

Polypill
What do you know about polypill therapies?- for treating cardiovascular disease?
Are you familiar with these?
What do you think about using a polypill for secondary prevention? Do you think it's feasible?
What would be the difficulties (if any) with using polypill for secondary prevention?
If a polypill became available for secondary prevention, is it something you would consider using?
